# Changes in bacterial and archaeal communities during the concentration of brine at the graduation towers in Ciechocinek spa (Poland)

**DOI:** 10.1007/s00792-017-0992-5

**Published:** 2017-12-19

**Authors:** Agnieszka Kalwasińska, Edyta Deja-Sikora, Aleksandra Burkowska-But, Attila Szabó, Támas Felföldi, Przemysław Kosobucki, Arkadiusz Krawiec, Maciej Walczak

**Affiliations:** 1 Department of Environmental Microbiology and Biotechnology, Faculty of Biology and Environmental Protection, Nicolaus Copernicus University in Toruń, Lwowska 1, Toruń, Poland; 2 Centre of Modern Interdisciplinary Technologies, Nicolaus Copernicus University in Toruń, Wileńska 4, Toruń, Poland; 3 Department of Microbiology, Eötvös Loránd University, Pázmány Péter stny. 1/c, H-1117 Budapest, Hungary; 4 Department of Food Analysis and Environmental Protection, Faculty of Chemical Technology and Engineering, UTP University of Science and Technology, Seminaryjna 3, Bydgoszcz, Poland; 5 Department of Geology and Hydrogeology, Faculty of Earth Sciences, Nicolaus Copernicus University in Toruń, Lwowska 1, Toruń, Poland

**Keywords:** Brine, Archaeal community, Bacterial community, Halophiles

## Abstract

This study evaluates the changes in bacterial and archaeal community structure during the gradual evaporation of water from the brine (extracted from subsurface Jurassic deposits) in the system of graduation towers located in Ciechocinek spa, Poland. The communities were assessed with 16S rRNA gene sequencing (MiSeq, Illumina) and microscopic methods. The microbial cell density determined by direct cell count was at the order of magnitude of 10^7^ cells/mL. It was found that increasing salt concentration was positively correlated with both the cell counts, and species-level diversity of bacterial and archaeal communities. The archaeal community was mostly constituted by members of the phylum Euryarchaeota, class Halobacteria and was dominated by *Halorubrum*-related sequences. The bacterial community was more diverse, with representatives of the phyla Proteobacteria and Bacteroidetes as the most abundant. The proportion of Proteobacteria decreased with increasing salt concentration, while the proportion of Bacteroidetes increased significantly in the more concentrated samples. Representatives of the genera *Idiomarina*, *Psychroflexus*, *Roseovarius*, and *Marinobacter* appeared to be tolerant to changes of salinity. During the brine concentration, the relative abundances of *Sphingobium* and *Sphingomonas* were significantly decreased and the raised contributions of genera *Fabibacter* and *Fodinibius* were observed. The high proportion of novel (not identified at 97% similarity level) bacterial reads (up to 42%) in the 16S rRNA gene sequences indicated that potentially new bacterial taxa inhabit this unique environment.

**Electronic supplementary material:**

The online version of this article (10.1007/s00792-017-0992-5) contains supplementary material, which is available to authorized users.

## Introduction

Salinity is an environmental factor which varies across natural aquatic systems due to both differences in the ratio of precipitation to evaporation and the input of dissolved ions from an area of land where precipitation collects and drains off into a common outlet. Concentrated salt solutions (brines) occur widely in the natural form of coastal lagoons, salt or soda lakes, deep-sea brines, groundwater, as well as in the form of salterns or saltworks of anthropogenic origin. Microorganisms adapted to life at high salt concentrations are found in all three domains of life and use different strategies of haloadaptation (Ma et al. 2010; Zajc et al. 2013; Harding et al. 2016).

The influence of salinity on bacterial and archaeal community composition in saline systems with rapid changes in salinity such as estuaries (Campbell and Kirchman 2013; Crump et al. 2004; Moss et al. 2006), soda pans (Szabó et al. 2017), and coastal solar salterns (Casamayor et al. 2002; Dillon et al. 2013), as well as in habitats with stable salinity conditions, characterized by a slow evolution from freshwater to saline (Felföldi et al. 2016; Máthé et al. 2014; Wu et al. 2006; Xing et al. 2009) has been investigated. These studies have definitively shown that salinity is one of the major factors controlling microbial abundance and diversity.

Groundwater aquifers with high temporal and horizontal stability are extreme habitats that contain microorganisms such as bacteria, archaea or fungi at low cell densities (Beyer et al. 2014). The knowledge on the diversity and functions of microbial communities in saline groundwater is limited. In the present study, we describe changes in bacterial and archaeal communities during the concentration of brine taken from deep subsurface environment (aquifer within Jurassic deposits, 405 m deep) and subsequently processed in the system of graduation towers.

A graduation tower is a unique structure used to produce salt which removes water from a saline solution by evaporation increasing the concentration of mineral salts. The tower consists of a wooden wall-like frame stuffed with bundles of brushwood (typically *Prunus spinosa* L.) splitting brine into droplets. The salt water runs down the tower and partly evaporates, and then it is discharged into the salt works. The maximal salt concentration which is practicable to obtain in a temperate climate zone is 27% and the optimal conditions for the process are sunny weather, temperature above 20 ℃, and a gentle wind. Under such favorable conditions, the maximal salt concentration can be achieved within 3 days. Both graduation towers and salterns, which are usually fed by seawater or saltwater springs, are used to produce salt through evaporation of water from a saline solution. Multi-pond solar salterns consist of a series of interconnected ponds with increasing salinity reaching sodium chloride saturation level (Boujelben et al. 2012, Gomariz et al. 2015), which is significantly higher than in the system of graduation towers. However, in case of graduation towers, the process of brine concentration may be faster, as the volume of saline water passing through is much lower (approximately 15,000 m^3^). Therefore, the changes in salinity observed in the graduation towers may be more rapid, compared to the system of solar salterns.

Solar salterns found worldwide, on the coasts of tropical and subtropical areas, harbor dense communities of extremely halophilic organisms generally dominated by archaea and characterized by low species richness and short food chains (Antón et al. 2000; Burns et al. 2004; Çinar and Mutlu 2016; Oren et al. 1996; Trigui et al. 2011). Contrary to them, the graduation towers can be found only in a few European spas (Poland, Germany, Austria) where they are used mainly as outdoor inhalatoria (Burkowska-But et al. 2014). The currently described community structure in hypersaline environments like solar salterns is that the square archaeon *Haloquadratum walsbyi,* the bacteroidete *Salinibacter ruber* and nanohaloarchaea are predominant members at the highest salt concentration, while more diverse archaeal and bacterial taxa are observed in habitats with intermediate salinities (Ventosa et al. 2015).

The present study is the first attempt to reveal the influence of salinity on bacterial/archaeal community composition and diversity in the artificial system of graduation towers with rapid dynamic salinity conditions fed by underground brine taken from Jurassic deposits and to identify bacterial taxa sensitive or tolerant to changes in water salinity.

## Materials and methods

### Study area

**Fig. 1:**
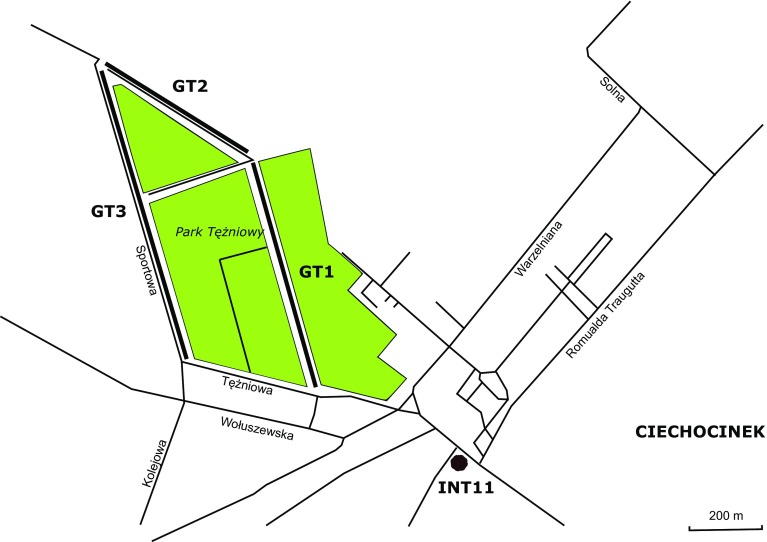
Outlook of the sampling sites

**Fig. 2:**
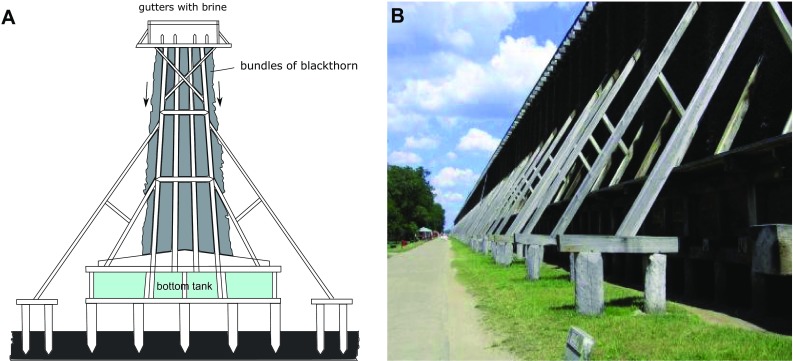
A graduation tower: a schematic diagram (**a**) arrows indicate the brine flow; photo (**b**)

There are three graduation towers (Fig. 1) in the center of Ciechocinek (mid-northern Poland coordinates: 52°52′58.2″N 18°47′13.8″E). The two largest towers were built in 1827–1828 and the third in 1859. Their total length is 1.7 km (Fig. 2). Their frames were made of oak and pine. Pumps raise brine from a borehole up to 15 meters above the ground level to the gutter on the upper platform. Borehole INT11 is located 400 m SE from the first graduation tower and its cover is in the shape of a mushroom and also acts as a fountain. The depth of the borehole drilled in 1909–1911 is 405 m [middle Jurassic deposits (Krawiec 2009)]. The saline water originates from meteoric waters of warm pre-Quaternary climates and also cold Quaternary climates with salinity gained from leaching numerous Zechstein salt diapirs (Krawiec 1999; Zuber and Grabczak 1991; Zuber et al. 2007). The temperature of the brine is 15 ℃, the concentration of total dissolved solids is 42 g/L and its redox potential is -11 mV (according to the Archive of the Spa, unpublished). The brine is concentrated at the three subsequent graduation towers (GT1–GT3 connected in series) during its way down through blackthorn branches to the lower gutters and then to the bottom tanks at the foot of each tower. Water from the tank GT1 is transferred directly on the top of the GT2 tower and water from the tank GT2 is transferred on the top of GT3 tower. Then the brine is discharged into the salt works. Graduation towers are used not only for the production of salt but also the microclimate around the area close to the towers acts as an inhalatorium or air filter.

### Brine samples

Brine samples were collected at four sampling sites in September 2015 from a wellhead (INT11) and after the concentration of the brine from the bottom tanks at the foot of the three subsequent graduation towers GT1–GT3 (Fig. 1). Samples (10 l) were collected using sterile glass bottles. Prior to the sampling, stagnant water from the borehole was pumped out and valve outlet was flame-sterilized. Bottles were completely filled with water avoiding air bubbles, then tightly sealed and transported directly to the laboratory in a portable ice bag at 4 ℃. All analyses started within 2 h after sample collection.

### Chemical analyses of brine samples

Total dissolved solids were measured by gravimetric method in 180°C and expressed in % as salinity. Dissolved oxygen was measured with a portable dissolved oxygen meter HI9143, Hanna Instruments (Michigan, USA), pH was determined with an Elmetron pH-meter (Zabrze, Poland) coupled with H^+^ (EPP-1) and Cl^−^ electrodes (Detektor, Poland) according to Knauss et al. (1990). Concentration of total carbon (TC) total organic/inorganic carbon (TOC, TIC) total nitrogen (TN) and ammonia nitrogen (NH_4_–N) was determined as described in Kalwasińska et al. (2015). All parameters were measured in triplicates.

### Enumeration of microorganisms

For determination of the total microbial cell counts, a 10-mL aliquot of each sample (in 3 replicates) was immediately fixed with formaldehyde at a final concentration of 4% upon collection. Following an overnight incubation at 4 °C, the fixed samples were filtered through black polycarbonate filters (0.22-µm pore size; Millipore, Bedford MA) and stained with acridine orange (final concentration 0.005 g/L) for 3 min. Microbial cells were counted with an epifluorescence microscope (Nikon Eclipse E200, Japan) at 1000× magnification on 10 randomly selected fields.

### Primers design

For next-generation DNA sequencing (NGS) with an Illumina MiSeq platform amplicons of the 16S ribosomal RNA gene were prepared with two pairs of primers used in two rounds of PCRs. Primers were designed in such a way to add unique barcodes (MID multiplex identifier) and sequencing adapters (P5 and P7) to the products. Primers of the first-round PCR contained universal sequences complementary to bacterial (357F: CCT ACG GGA GGC AGC AG and 786R: ACC AGG GTA TCT AAW CC; (Alm et al. 1996; Gołębiewski et al. 2014)) and archaeal (513F: GGT GYC AGC CGC CGC GGT AA and 915R: GTG CTC CCC CGC CAA TTY CT) 16S rRNA genes. Universal sequences were proceeded by M13 sequences (at the 5′ ends): GTT TTC CCA GTC ACG AC for F primers and CAG GAA ACA GCT ATG AC for R ones. M13 sequences were used as priming sites in the second round of PCR that was performed with other primers containing from the 3′ ends: M13, multiple identifiers (MID), and P5 (AAT GAT ACG GCG ACC ACC GAG ATC TAC AC) or P7 (CAA GCA GAA GAC GGC ATA CGA GAT) adapters to the MiSeq flow cell.

HPLC-purified PCR primers of the first round were used as custom sequencing primers for read 1 and read 2. Additionally, custom i5 index read primers (786R_i5: GGW TTA GAT ACC CTG GTC CGA CGA CGA CGG TCA TAG CTG TTT CCT G and 915R_i5: AGR AAT TGG CGG GGG AGC ACG TCA TAG CTG TTT CCT G) were used during sequencing.

### Extraction of microbial community DNA

Water samples (250 mL) were filtered through sterile polycarbonate filter membranes (0.22 µm pore size Millipore). Fragmented filters were put into the tubes and used for total genomic DNA isolation using a method described by Zhou et al. (1996). DNA concentration was measured with Qubit 2.0 (Invitrogen) using Qubit dsDNA HS Assay Kit (Thermo Fisher Scientific).

### Preparation of 16S rDNA amplicon libraries

Fragments of bacterial and archaeal 16S rDNA sequences were generated by the following PCR protocol. Reaction mixtures contained: 1 µL of metagenomic DNA, 0.2 mM dNTPs, 0.25 µM primers of the first round, 1 U High Fidelity Phusion Polymerase, and 1× buffer with 1.5 mM MgCl_2_ (Thermo Fisher Scientific). Thermal profile of the reaction contained: initial denaturation at 98 °C for 30 s; 30 cycles of − 98 °C for 10 s, 55 °C (bacterial primers)/62.5 °C (archaeal primers) for 15 s, 72 °C for 10 s; and final elongation at 72 °C for 5 min. The concentration of product was measured with Qubit 2.0 and Qubit dsDNA HS Assay Kit. 50 pg of amplicons from each reaction were used for another PCR round containing: 0.2 mM dNTPs 0.25 µM second-round primers 1 U Taq Polymerase and 1x buffer with 1.5 mM MgCl_2_ (Thermo Fisher Scientific). Cycling conditions were following: initial denaturation at 95 °C for 3 min; 15 cycles of − 95 °C for 30 s, 54 °C for 20 s, 72 °C for 30 s; and final elongation at 72 °C for 5 min. Then the concentrations of 16S rDNA amplicons were measured as mentioned before.

Bacterial and archaeal libraries were created by pooling equal quantities of amplicons. Libraries were double purified with Agencourt AMPure XP (Agencourt Bioscience) and evaluated using a model 2100 Bioanalyzer (Agilent Technologies) with a High Sensitivity DNA Analysis Kit (Agilent Technologies).

### Library quantification and sequencing

16S rRNA gene amplicons were quantified with the KAPA DNA Library Quantification Kit (KAPA Biosystems). Reaction mixtures were prepared according to the manufacturer’s protocol. Quantification run was performed on a Roche LightCycler 480 System. Bacterial and archaeal libraries were pooled in equimolar ratio and diluted to obtain a single 6 pM sequencing library per sample with 10% PhiX DNA. Sequencing was performed on a MiSeq platform (Illumina) using a MiSeq Reagent Kit v3 (600 cycles). HPLC-purified custom sequencing primers were mixed with Illumina primers. Demultiplexing of indexed reads was performed with the MiSeq software.

### Bioinformatics analysis

Sequence reads were processed using mothur v1.35 (Schloss et al. 2009) as recommended by the MiSeq SOP page (http://www.mothur.org/wiki/MiSeq_SOP downloaded at 25/01/2016) (Kozich et al. 2013). Sequences obtained with the Archaea- and Bacteria-specific primers were assorted based on the alignment using the ARB-SILVA SSU NR99 reference database—SILVA release 123 (Quast et al. 2013). Chimera detection was performed with UCHIME (Edgar et al. 2011). Besides chimeric sequences, singleton reads were also removed (using mothur’s ‘split.abund’ command) according to Kunin et al. (2010). Taxonomic assignments were made against SILVA release 123 applying a minimum bootstrap confidence score of 80%. Operational taxonomic units (OTUs) were assigned at 97% similarity threshold level as suggested by Tindall et al. (2010) for prokaryotic species delineation. Raw sequence reads were deposited in NCBI SRA under BioProject ID PRJNA383523.

### Calculating the richness and diversity of the total bacterial and archaeal community

Species richness Chao1 (Chao 1984) and ACE (Chao and Lee 1992) indices were assessed at the 0.03 dissimilarity level (representing a threshold value for bacterial species according to Tindall et al. 2010) with the mothur software (Schloss et al. 2009) for thousand-fold randomized subsamples of 3163 sequences/sample for Bacteria and 967 sequences/sample for Archaea. Bray–Curtis (Bray and Curtis 1957) and Morisita-Horn (Horn 1966) dissimilarities were calculated for all the pairs of samples using vegan (Oksanen et al. 2017) in R v 3.3.1 and visualized via corrplot in R (Wei and Simko 2016). Venn diagrams were generated from the shared OTU (operational taxonomic unit) table using a web interface for creating Venn diagram from Gent University (http://bioinformatics.psb.ugent.be/webtools/Venn/).

### Statistical analysis

The differences between values of microbial densities and species richness/diversity estimators were tested with one-way ANOVA and the pairwise differences were checked with Tukey HSD test (Statistica 97 ver. 6.0). Spearman’s correlation coefficients between physico-chemical parameters of the brines and bacterial/archaeal diversity were performed in R using Hmisc package (Spearman’s correlation in rcorr; (Harrell 2016)). Principal component analysis (PCA) was carried out using FactoMineR in R (Lê et al. 2008). Principal Coordinates Analysis (PCoA) was performed using Vegan (Oksanen et al. 2017). The envfit function was used to link microbial community composition (OTUs at the 0.03 level) to the environmental variables. Environmental data were standardized using z-scoring and correlation matrix of environmental variables was calculated prior to PCA and PCoA. Parameters showing strong positive correlation (≥0.8) were reduced to one variable representing both of them and expressed on the diagram as TC/TIC.

## Results

### Brine characteristics

Physicochemical characteristics of brines are presented in Table 1. Although the samples had quite similar neutral pH values (between 6.6 and 7.4), they differed much regarding the concentration of salt. Brine from the borehole (INT11) feeding the first graduation tower had the lowest salinity (5.1 ± 0.02%), while brine from the last sampling site (GT3) had the highest salinity (26.7 ± 1.75%). Groundwater from the borehole (INT11) did not contain dissolved oxygen (DO), but after passing through the system of graduation towers, the concentration of dissolved oxygen varied between 4.13 ± 0.07 ppm and 1.14 ± 0.04 ppm. With the increase in salt concentration, a clear decline in TIC values in the samples was observed (from 61.7 to 26.5 mg/L), while TOC starting from GT1 to GT3 showed a rising trend (from 48.2 to 90.4 mg/L). Nutrient concentrations varied only slightly across sites. The lowest concentration of TN was recorded in INT11 (7.7 mg/L) and the highest in sample GT1 (10.6 mg/L). The concentration of NH_4_–N was very similar at the sites (from 0.035 to 0.040 mg/L). TC and TIC were positively and significantly correlated (*R*2 = 0.8 *p* < 0.05; data not shown).

**Table 1:** Chemical properties of the brines from Ciechocinek

Sample code	Salinity (%)	DO (ppm)	pH	TC (mg/L)	TOC (mg/L)	TIC (mg/L)	TN (mg/L)	NH_4_–N (mg/L)	Density of microorganisms (cells/mL, 10^7^)
INT 11	5.1 ± 0.02	< LOD	7.05 ± 0.02	141.0 ± 2.87	68.3 ± 3.17	61.7 ± 1.56	7.7 ± 0.02	0.035 ± 0.002	2.25 ± 1.05
GT1	8.7 ± 0.9	4.13 ± 0.07	6.62 ± 0.03	88.23 ± 1.11	48.2 ± 1.24	40.0 ± 0.74	10.6 ± 0.02	0.039 ± 0.001	2.96 ± 0.26
GT2	16.4 ± 1.5	1.92 ± 0.03	7.39 ± 0.04	91.25 ± 2.13	61.7 ± 2.08	29.6 ± 0.89	8.8 ± 0.01	0.037 ± 0.002	3.35 ± 0.84
GT3	26.7 ± 1.7	1.14 ± 0.04	7.15 ± 0.03	116.90 ± 1.89	90.4 ± 1.72	26.5 ± 0.90	9.5 ± 0.02	0.040 ± 0.004	5.47 ± 0.46

Each value was calculated based on the results of three parallel measurements

*DO* dissolved oxygen, *TC* total carbon; *TOC* total organic carbon, *TIC* total inorganic carbon, *TN* total nitrogen, *NH*_*4*_ *–N* ammonia nitrogen, *LOD* limit of detection, *INT11* sample collected from the borehole, *GT1–GT3* bottom tank samples collected from the three graduation towers

The principal component analysis of physicochemical data showed the distinct dispersion of the sampling sites (Fig. S1). The first and the second PCA axis taken together explained 91.5% of the observed variation (49.9 and 41.6%, respectively). The brine from the sampling site GT3 was the most distinct sample and it was characterized by the highest salinity. Due to a low sample size, the significance of the environmental factors was not tested.

### Abundance of microorganisms

The microbial cell density determined by direct cell count ranged from 2.24 ± 1.05 × 10^7^ cell/mL (sample with the lowest salt concentration) to 5.47 ± 0.46 × 10^7^ cells/mL (the most saline sample Table 2) and was positively correlated with EC (*R*2 = 0.82 *p* < 0.01) and negatively correlated with TIC (*R*2 = − 0.82 *p* < 0.01). The total number of microbes at site GT3 differed significantly from the values measured at other sites (*p* < 0.01).

**Table 2:** NGS data statistics of the brine samples from Ciechocinek

	Sample code	Number of sequences	Good’s coverage (%)	Sobs	ACE	Chao 1	Inverse Simpson’s (1/*D*)	Shannon’s diversity (*H*′)	Shannon’s evenness (*E*)
Bacteria	INT 11	6096	99.6	59 ± 2	69 ± 6	65 ± 5	5.42 ± 0.11	2.31 ± 0.02	0.57 ± 0.006
	GT1	5495	99.7	96 ± 1	102 ± 2	98 ± 1	9.25 ± 0.10	3.10 ± 0.01	0.68 ± 0.002
	GT2	5046	99.1	162 ± 2	182 ± 5	173 ± 5	7.74 ± 0.11	3.01 ± 0.02	0.59 ± 0.003
	GT3	3999	99.7	127 ± 1	131 ± 1	128 ± 1	14.13 ± 0.01	3.33 ± 0.01	0.69 ± 0.001
Archaea	GT1	989	99.9	8 ± 1	9 ± 1	8 ± 1	4.56 ± 0.1	1.63 ± 0.01	0.79 ± 0.001
	GT2	5039	99.9	73 ± 4	109 ± 21	99 ± 14	10.94 ± 0.53	2.98 ± 0.04	0.70 ± 0.008
	GT3	3448	99.7	68 ± 3	107 ± 23	89 ± 12	11.94 ± 0.45	2.98 ± 0.04	0.71 ± 0.007

The number of OTUs found in the subset of sequences was normalized to the sample with the lowest sequence count

### Bacterial and archaeal community structures

After quality filtering, 20,636 and 9476 high-quality bacterial and archaeal reads were acquired, respectively, from a total of 4 brine samples (Table 2). The communities were moderately sampled, indicating that more reads would be required to capture all the diversity, especially in case of sampling sites GT2 and GT3 (Fig. S2). Clustering at 97% similarity level resulted in total 328 bacterial and 132 archaeal OTUs. Both the observed (Sobs) and estimated total richness (ACE Chao1) of bacterial and archaeal communities increased with the increasing salt concentration and were the highest in sample GT2 (Table 2). However, in sample GT3, which had the highest salinity, these values were in between the values of samples GT1 and GT2. In case of bacterial and archaeal diversity (i.e. Shannon and Inverse Simpson’s) values were the highest in the most saline sample (GT3). Regression analysis showed that the salinity positively correlated with all estimators of bacterial species richness and diversity (Table 3), and with archaeal diversity indices. Another environmental factors significantly correlating with mentioned above bacterial estimators were pH and TOC (positive correlation) as well as TIC (negative correlation). In case of archaeal communities a strong negative correlation between archaeal diversity and TIC/TN was found, as well as with DO. However, due to the low number of samples, such results should be treated cautiously in spite of their high statistical significance.

**Table 3:** Spearman’s correlation coefficients between physico-chemical parameters of the brines and bacterial/archaeal richness and diversity values

	pH	Salinity	DO	TOC	TIC	TN	NH_4_–N
Bacteria							
*S*_obs_	0.76**	0.76**	0.40	0.01	− 0.72**	0.22	0.36
*H*′	0.67*	0.69*	− 0.47	0.73**	− 0.78**	− 0.28	− 0.11
1/*D*	0.73**	0.73**	− 0.42	0.73**	− 0.81**	− 0.24	− 0.06
Archaea							
*S*_obs_	0.75*	0.60	-0.50	0.40	− 0.50	− 0.85**	− 0.27
*H*′	0.61*	0.68*	− 0.85**	0.61	− 0.85**	− 0.85**	− 0.58
1/*D*	0.57	0.72*	− 0.92**	0.72	− 0.92***	− 0.77*	− 0.52

*p < 0.5, ** *p* < 0.01, *** *p* < 0.001

**Fig. 3:**
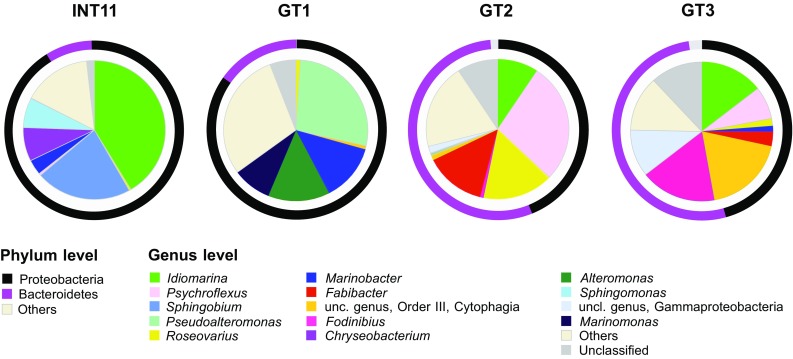
Phylum and genus level distribution of NGS reads within the domain Bacteria in the brine samples from Ciechocinek

An analysis of the distribution of the most abundant bacterial phyla revealed that the brines from the system of graduation towers were dominated by Proteobacteria and Bacteroidetes (Fig. 3). The contribution of Proteobacteria decreased with increasing salt concentration (R Spearman = − 0.95 *p* < 0.05). Contrary to this, the proportion of Bacteroidetes increased significantly in more concentrated samples (R Spearman = 0.89 *p* < 0.001). The most abundant classes across the samples were Gammaproteobacteria and Alphaproteobacteria followed by Flavobacteriia. These two proteobacterial classes taken together were dominating in INT11 and GT1 (> 80% of the communities), while their levels were much lower in GT2 and GT3 (< 45%). The water that passed through the second graduation tower had the highest relative abundance of Flavobacteriia and increased level of Cytophagia (35 and 16%, respectively). However, the highest representation of Cytophagia (40.2%) was detected in the sample GT3. Detailed analysis of the bacterial community composition at the genus level showed that the percentage of unclassified bacterial sequences (2, 6, 12 and 42% of total bacterial reads) increased with salinity. Many of these sequences were only classified down to class or family level, which means that new phylotypes exist in the studied brines. Shannon’s evenness ranged from 0.57 to 0.69 (for INT11 and GT3, respectively), which suggested that a few bacterial species dominated the assessed microbiomes. The bacterial community of the groundwater (INT11) was dominated by *Idiomarina*, *Sphingobium*, *Sphingomonas*, and *Chryseobacterium* before entering the GT1–GT3 system (Fig. 3, Table S1). The flow of the groundwater brine through the first graduation tower resulted in a loss of the groundwater-specific genera mentioned above. We observed the raised level of *Marinobacter*, *Roseovarius*, *Pseudoalteromonas*, *Alteromonas*, and *Marinomonas* in the GT1 sample. Interestingly *Roseovarius* reached the highest level in the GT2 sample whereas the proportions of the other genera found in GT1 strongly decreased when the concentration of the brine raised in the next two graduation towers. Generally, GT2 and GT3 communities differed markedly from the GT1. We noticed the resurgence of *Idiomarina* and co-occurrence of *Psychroflexus Fabibacter*, *Fodinibius*, unclassified Gammaproteobacteria, and Cytophagia in the GT2-GT3 communities. However, *Psychroflexus*, *Fabibacter*, and *Roseovarius* were dominant in the GT2 sample, while *Fodinibius*, *Idiomarina*, and two unclassified genera belonging to Gammaproteobacteria and Cytophagia were the most abundant in the GT3 sample.

**Fig. 4:**
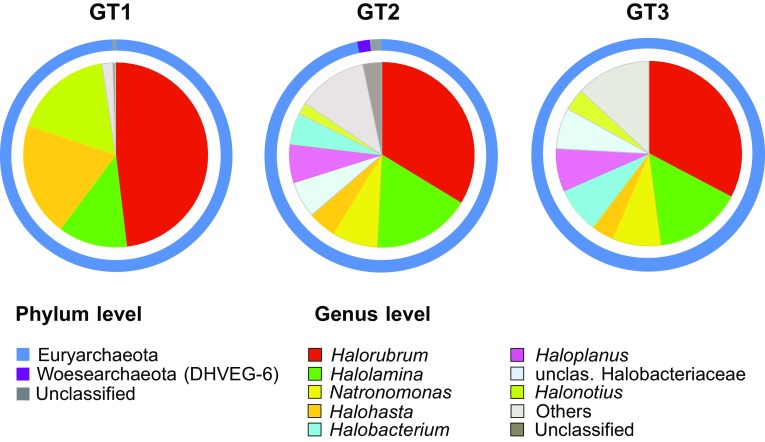
Phylum and genus level distribution of NGS reads within the domain Archaea in the brine samples from Ciechocinek

Archaeal reads were detected in all brine samples except INT11. They were dominated by members of the phylum Euryarchaeota (Fig. 4), class Halobacteria. Only a small percentage of reads at the site GT2 (1.8%) belonged to unclassified members of the phylum Woesearcheota. Shannon’s evenness of archaeal assemblages varied between 0.70 and 0.79 (for GT2 and GT1, respectively) which suggested that a few archaeal species dominated. The most abundant genera in all samples were *Halorubrum Halohasta Halonotius* and *Halolamina* (Fig. 4, Table S1). The proportion of the first three genera markedly decreased in more concentrated brines. On the other hand sequences related to *Natronomonas Halobacterium Haloplanus* and unclassified Halobacteriaceae increased and co-occurred in the GT2–GT3 communities. Interestingly, GT2 and GT3 communities differed significantly from GT1 and were characterized by a very similar pattern of distribution of the most abundant genera.

**Fig. 5:**
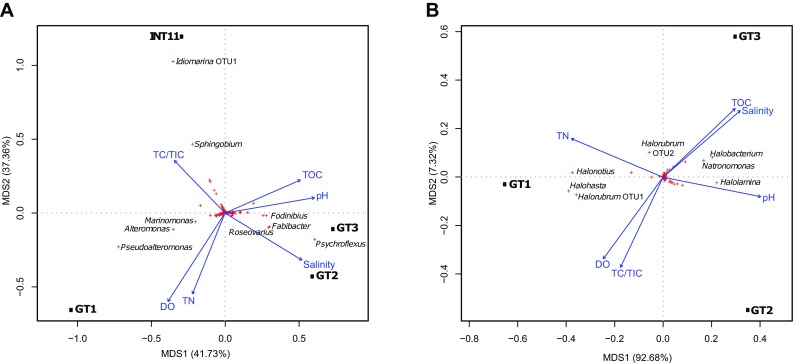
Principal coordinates analysis (PCoA) of the bacterial (**a**) and archaeal (**b**) communities based on the Bray–Curtis distance matrix

**Fig. 6:**
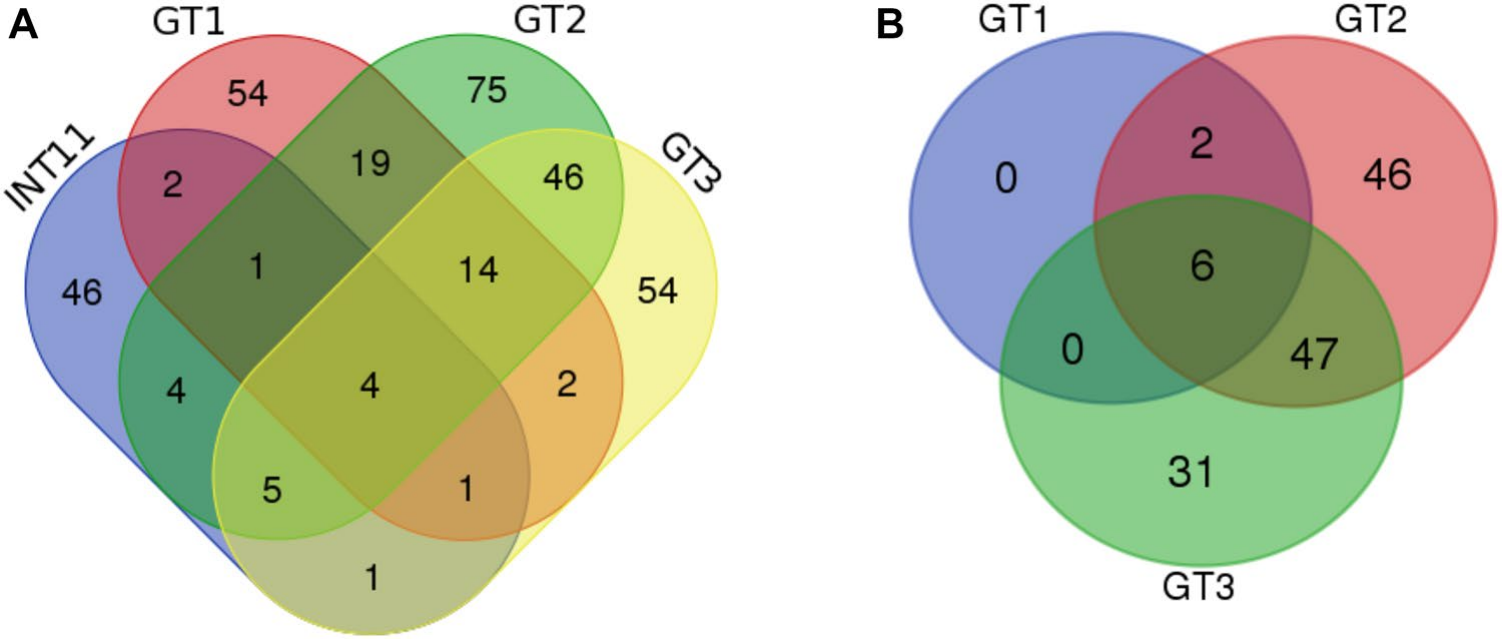
Venn diagrams of shared bacterial (**a**) and archaeal (**b**) OTUs at 0.03 dissimilarity level

Incidence- as well as abundance-based distances: Bray–Curtis (B–C) and Morisita–Horn (M–H), respectively, were used to calculate dissimilarities between the communities in the samples and community similarity heatmaps were generated (Fig. S3 and Fig. S4). In case of archaeal communities the B–C similarities were smaller than the M–H ones indicating that the structures of the abundant OTUs were more similar than the rare OTUs community structures. Furthermore, the values of B–C and M–H similarities were higher in case of archaeal communities compared to bacterial communities. In other words, bacterial communities were more dissimilar among sites than archaeal communities. GT2 and GT3 appeared to be the most similar samples while INT11 and GT1 showed little similarity to the others. In case of archaeal communities, samples GT2 and GT3 had almost the same taxonomic composition and GT1 differed from them. A similar pattern of separation of the bacterial and archaeal community composition was shown by PCoA biplots (Fig. 5). The results of the community “overlaps” depicted as Venn diagrams (Fig. 6) corroborated with the analyses presented above. Only 4 bacterial and 6 archaeal OTUs were shared by all four samples. The most abundant shared bacterial OTUs at 0.03 clustering level were *Psychroflexus* (OTU2), *Idiomarina* (OTU5), and *Roseovarius* (OTU6). Among archaeal sequences the most abundant shared OTUs were *Halorubrum* (OTU1, OTU2), *Halolamina* (OTU4), *Halohasta* (OTU6), and *Halonotius* (OTU7).

## Discussion

The densities of microorganisms in brines were relatively high on average 10^7^ cells/mL. They were similar to those detected in the solar salterns throughout the world (Oren et al. 1996; Antón et al. 2000; Burns et al. 2004; Trigui et al. 2011; Máthé et al. 2014; Andrei et al. 2015; Gomariz et al. 2015; Felföldi et al. 2016; Çinar and Mutlu 2016). Hypersaline environments of greater abundance of microorganisms (more than 10^8^ cells/mL) are also known (Guixa-Boixareu et al. 1996; Ochsenreiter et al. 2002). This high density of cells in many hypersaline environments is thought to be a result of the lack of predation and the presence of high nutrient levels (Oren 2002). The total number of microorganisms in the sample from the borehole (INT11) feeding the graduation towers was two or three orders of magnitude higher than the densities recorded in the case of many other terrestrial subsurface environments (10^4^–10^5^ cells/mL) (Beaton et al. 2016; Pedersen and Ekendahl 1990; Walczak et al. 2017); however, the total number of microorganisms close to 10^4^ cells/mL may be the limit value in groundwaters with a very low concentration of organic carbon (Fry et al. 1997; Lovley and Goodwin 1988). The mean concentration of TOC in natural European groundwaters is relatively low − 2 mg/L with minimum and maximum values below 0.5 and above 50 mg/L, respectively (Gooddy and Hinsby 2008). The relatively high TOC concentrations in the investigated brines from 48 to 90 mg/L may explain the high abundance of microorganisms, presumably heterotrophic microorganisms present in the samples.

A widely held ecological tenet states that a more extreme environment is expected to maintain lower species diversity (Frontier 1985). However, this is not the case here. Both the observed, and estimated bacterial species richness and diversity in the studied samples increased along the salinity gradient (5–27%). Similar findings were recorded by other researchers in solar salterns (Casamayor et al. 2002; Øvreås et al. 2003), but in the ponds with the highest degree of salt concentration (around 35–37% crystallizer ponds), the richness of taxa at the genus level was usually reduced, compared with ponds of lower salinity and often resulted in a few dominant phylotypes (Çinar and Mutlu 2016; Ventosa et al. 2015). Contrary to the observed significant positive correlation between bacterial species richness/diversity and salinity, there was no such statistical relationship in case of archaeal communities in the investigated brines. The archaeal communities, especially GT2 and GT3, were more similar among sites than the bacterial ones. Similar observation was made by Hoshino et al. (2011), and Purkamo et al. (2016) who compared bacterial and archaeal communities in subsurface environments.

Archaeal communities of the brines from the system of graduation towers in Ciechocinek were dominated by members of Euryarchaeota (Halobacteriaceae), which is in line with reports of other authors studying microbial diversity in environments with a broad range (3–37%) of salinity gradient (e.g., Ventosa et al. 2015). The comparative analysis of prokaryotic communities of salterns of a medium salinity (20–27%) in Turkey, based on high-throughput 16S rDNA amplicon sequencing data, and two medium concentrators in Tunisia and Spain (salinity 20–28 and 18–29%, respectively), based on DGGE present similar findings, with all archaeal sequences assigned to the class Halobacteria and family Halobacteriaceae (Çinar and Mutlu 2016; Boujelben et al. 2012; Gomariz et al. 2015). Nevertheless, we observed that the GT2–GT3 (salinity 16 and 27%, respectively) pattern of taxa distribution differed from the less concentrated GT1 (9%) and comprised quite evenly distributed sequences related to *Natronomonas*, *Halohasta, Halobacterium*, and *Haloplanus*. The similar structure of archaeal assemblages may manifest a stabilization of the community composition at the genus level at the elevated levels of salinity.

Interestingly, the study of the graduation towers microbiome revealed considerable novelty. There was no evidence of the presence of sequences belonging to the genus *Haloquadratum* that usually dominates microbial communities in hypersaline waters, close to or above saturation, but is also present in medium salinity brines (Dyall-Smith et al. 2011; Ventosa et al. 2015; Podell et al. 2014; Gomariz et al. 2015). The most abundant archaeal reads in our samples belonged to the hyperhalophilic genera *Halorubrum* and *Halolamina*. Representatives of the genus *Halorubrum* dominated over *Haloquadratum* in the two Turkish salterns Fadlum and Tuzlagözü (salinity 27 and 20%, respectively, Çinar and Mutlu 2016), as well as in the Spanish saltern SS19 Santa Pola with medium salinity (19%, Ghai et al. 2011). The second more abundant archaeal genus in brines from graduation towers in Ciechocinek was *Halolamina* while in the case of Turkish salterns sequences related to this genus constituted only a minor fraction of the archaeal dataset (Çinar and Mutlu 2016). Representatives of the genus *Halolamina* were first isolated from the Taibei marine solar saltern, China (Cui et al. 2011) and were also found in purified solar salt and crystallizing pond of Gomso solar saltern, Republic of Korea (Cha et al. 2014; Park et al. 2012). Therefore, their presence in the hypersaline environment studied here is explainable.

It should also be pointed that archaeal reads were not detected in the sample taken from the borehole (INT11), suggesting that the representatives of the domain Archaea may not be present there or were present at low abundance. It would not be surprising in case of Halobacteriaceae which prevailed in other samples. Members of this family are thought to be obligate halophiles (Oren 2008) and may be more demanding in terms of high salt concentration than the concentration found in the brine from the borehole. On the other hand, Archaea are more cosmopolitan in nature than was previously thought (e.g. DeLong 1992) and were also detected in a low-salt subsurface environment (Chaudhary et al. 2009; Elshahed et al. 2004; Fry et al. 2008).

Bacterial communities of the brines from the system of graduation towers in Ciechocinek were dominated by the phyla Proteobacteria and Bacteroidetes. The proportion of Proteobacteria (especially class Alphaproteobacteria) decreased with increasing salt concentration, while the proportion of Bacteroidetes increased significantly in the more concentrated samples (up to 27%), which is consistent with the data reported by other authors (Çinar and Mutlu 2016; Ventosa et al. 2015; Gomariz et al. 2015; Fernández et al. 2014). Single-cell genome analysis of some members of Bacteroidetes described by Gomariz et al. (2015) suggested a set of metabolic features which may be responsible for competitive advantages in hypersaline environments, such as polymer degradation capabilities, the presence of retinal-binding, light activated proton pumps and also arsenate reduction potential.

Our research demonstrated that a few bacterial taxa namely *Idiomarina*, *Psychroflexus*, *Roseovarius*, and *Marinobacter* appeared over a wide salinity range, i.e., 4.5–27% showing their broad ecological plasticity. Indeed their representatives have been isolated from a variety of environments starting from estuaries and seawater to hypersaline crystallizer ponds (Lee et al. 2015; Máthé et al. 2014; Park et al. 2016; Zhong et al. 2015). On the other hand, one of the possible microbial community responses to increasing salinity is the replacement of suboptimally adapted taxa by taxa better adapted to the given salinity conditions (Wu et al. 2006). This can clearly be seen, for instance, in the composition of the first two samples INT11 and GT1. *Sphingomonas* and *Sphingobium*-related sequences (Alphaproteobacteria) which were abundant in the underground brine of a lower salinity (4.5%) were replaced by *Alteromonas* and *Pseudoalteromonas*-related sequences (Gammaproteobacteria) in the more concentrated brine sample GT1 (9% NaCl). According to the physiological data on NaCl tolerance of particular bacterial strains available in LPNS database (List of Prokaryotic Names with Standing in Nomenclature; http://www.bacterio.net) *Sphingomonas* and *Sphingobium* show significantly lower salt tolerance (usually up to 1% NaCl) than *Alteromonas* and *Pseudoalteromonas* (up to 15–20% NaCl and 6–13% NaCl, respectively).

The gradual adaptation of the same taxa which is another possible response to increasing salinity may be particularly noticeable in case of *Salegentibacter* (OTU018). This genus encompasses both moderately halophilic and highly halotolerant bacteria (Ivanova et al. 2006; Yoon et al. 2008).

The relatively high percentage of novel bacterial reads in the more concentrated samples that reached 42% of total bacterial sequences at the genus level in GT3 is remarkable for the obtained dataset. This is a significantly higher level of novel reads than previously noted for solar salterns (Çinar and Mutlu 2016; Fernández et al. 2014; Podell et al. 2014), which indicate that potentially new bacterial taxa inhabit this unique environment. On the other hand, the number of novel archaeal reads obtained for the GT2 and GT3 datasets was significantly lower and did not exceed 10% of the total archaeal reads at the genus level. These results are comparable to those obtained for Turkish salterns of similar salinity (Çinar and Mutlu 2016) and significantly lower than that for the Spanish SS13 saltern (Fernández et al. 2014), where as much as 92% of archaeal sequences were not classified at genus level.

The genus *Salinibacter* comprising the most halophilic bacteria known and sharing many properties with halophilic Archaea such as *Haloquadratum* has been shown to be a dominant member of some crystallizer communities, e.g., Fadlum (Çinar and Mutlu 2016) or SS19 and SS37 in Santa Pola salterns (Ghai et al. 2011) and hypersaline lakes, e.g., Tyrrell (Australia), Aran-Bidgol (Iran) (Podell et al. 2014; Makhdoumi-Kakhki et al. 2012). In addition to *Salinibacter,* representatives of the genera *Salisaeta* and *Euhalothece* were among the dominant taxa in Turkish salterns Bingöl and Fadlum (salinity 25 and 27%, respectively) (Çinar and Mutlu 2016)). Although *Salinibacter* is an important part of the bacterial communities in hypersaline environments, the 16S rRNA gene reads related to it were not detected in our samples, nor in the Andean saltern described by Maturrano et al. (2006). The lack of genera *Salinibacter* and also *Salisaeta* in the system of graduation towers in Ciechocinek could be related with the lower levels of solar radiation and lower temperature, compared to the salterns and lakes where these organisms were detected. *Salinibacter* and presumably *Salisaeta* (Schneider et al. 2013) and also *Haloquadratum* are capable of phototrophic growth and lack of the sunshine in the bottom tanks from which the samples were taken may limit their growth in the multi-species competitive conditions. Instead in the most concentrated sample GT3, a considerable number of reads (~ 18% of total reads) were assigned to *Fodinibius.* Members of this genus were first isolated from a salt mine in the Yunnan province China (Wang et al. 2012) but they have never been found yet in abundance in hypersaline environments. Besides *Fodinibiu*s in the moderately saline GT2 sample the relatively high proportion of reads was classified as *Fabibacter* (~ 13% of total reads). Its representatives were isolated from samples of marine origin (Wong et al. 2015) and *F. halotolerans* showed moderate salt tolerance up to 12% NaCl.

Although the bacterial and archaeal communities were largely dominated by halotolerant and halophilic microorganisms in all the samples studied, there was a significant difference in the bacterial community structure between the samples. The distinct difference in the bacterial assemblages was most likely caused by salinity changes. The influence of salinity on microbial community composition in various water ecosystems was extensively investigated by other researchers (Jiang et al. 2007; Maturrano et al. 2006; Yang et al. 2016; Zhang et al. 2013). These studies have definitively shown that salinity is one of the most important factors controlling bacterial abundance diversity and metabolic activity exceeding the influence of temperature and pH (Lozupone and Knight 2007; Wang et al. 2011; Boujelben et al. 2012; Wu et al. 2006). Other factors such as nutrient concentration and organic matter composition gradient also cannot be neglected (Oren 2002).

Major physico-chemical environmental shifts from, e.g., anoxic into oxic conditions have a direct impact on community composition and richness of many organisms (Andrei et al. 2015; Herlemann et al. 2011; Máthé et al. 2014). This could be the second important factor affecting investigated microbiome of graduation towers. The most dramatic change may take place when brine from the borehole containing little or no oxygen enters the first graduation tower GT1 and after passing through it becomes saturated with atmospheric air. Oxygen delivery may affect bacterial and archaeal community structure of GT1 which is unique across the samples. The low solubility of oxygen in salt-saturated brines and the potentially high heterotrophic activity of the dense microbiota often results in a near-anaerobic conditions in the hypersaline environments (Oren 2017) and this can be the reason why GT3 bacterial community is more similar to INT11 community than to GT1.

## Conclusions

The microbial composition of the brines from the system of graduation towers was remarkable. The increasing salts concentration (4.5–27%) increased the cell counts and diversity of bacterial and archaeal communities. Bacterial communities were generally more diverse than archaeal communities. Both the archaeal and the bacterial community compositions were remarkably different from what was expected. The *Haloquadratum* and *Salinibacter*-related sequences which are characteristic for hypersaline environments were absent. A strong increase of the number of novel sequences with increasing salinity was observed indicating that potentially new bacterial taxa inhabit the unique environment of graduation towers.

## Electronic supplementary material

Below is the link to the electronic supplementary material.

**Table S.1** The most abundant bacterial and archaeal OTUs in brines from Ciechocinek INT11 – sample collected from the borehole, GT1-GT3 - bottom tank samples collected from the three graduation towers (DOCX 19 kb)

**Fig. S1** PCA of physical-chemical properties of brines from the system of graduation towers in Ciechocinek (PDF 27 kb)

**Fig. S2** Rarefaction analysis of sequences from the brines from Ciechocinek at 0.03 dissimilarity level; a - bacterial b - archaeal INT11 – sample collected from the borehole, GT1-GT3 – bottom tank samples collected from the three graduation towers (PDF 51 kb)

**Fig. S3** Bacterial community distance heatmaps. Heatmaps based on Bray-Curtis (A) and Morisita-Horn (B) at 0.03 dissimilarity level. Light blue - small distances (greater similarity), dark blue - greater distances (lower similarity) (PDF 21 kb)

**Fig. S4** Archaeal community distance heatmaps. Heatmaps based on Bray-Curtis (A) and Morisita-Horn (B) at 0.03 dissimilarity level. Light blue - smaller distances (greater similarity), dark blue - greater distances (lower similarity) (PDF 20 kb)
