# Supplementary material for: Changes in bacterial and archaeal communities during the concentration of brine at the graduation towers in Ciechocinek spa (Poland)
Source: Extremophiles. 2017 Dec 19;22(2):233–46. doi: 10.1007/s00792-017-0992-5 (PMC5847177; doi:10.1007/s00792-017-0992-5)
Supplement: Supplementary file 1 — Table S.1 The most abundant bacterial and archaeal OTUs in brines from Ciechocinek INT11 – sample collected from the borehole, GT1-GT3 - bottom tank samples collected from the three graduation towers (DOCX 19 kb) [file 792_2017_992_MOESM1_ESM.docx]

**Table S1** The most abundant bacterial and archaeal OTUs in brine samples from Ciechocinek

| OTU | Contribution to the microbial diversity (%) | | | | Taxonomy |
| --- | --- | --- | --- | --- | --- |
|  | INT11 | GT1 | GT2 | GT3 |  |
| Otu1 | 37.0 | 0.0 | 0.9 | 7.5 | Proteobacteria; Gammaproteobacteria; Alteromonadales; Idiomarinaceae; Idiomarina |
| Otu2 | 0.4 | 0.4 | 27.4 | 7.5 | Bacteroidetes; Flavobacteriia; Flavobacteriales; Flavobacteriaceae; Psychroflexus |
| Otu3 | 16.5 | 0.0 | 0.1 | 0.0 | Proteobacteria; Alphaproteobacteria; Sphingomonadales; Sphingomonadaceae; Sphingobium |
| Otu4 | 0.0 | 27.5 | 0.2 | 0.0 | Proteobacteria; Gammaproteobacteria; Alteromonadales; Pseudoalteromonadaceae; Pseudoalteromonas; |
| Otu5 | 4.4 | 0.1 | 7.9 | 6.5 | Proteobacteria; Gammaproteobacteria; Alteromonadales; Idiomarinaceae; Idiomarina |
| Otu6 | 0.5 | 0.8 | 16.2 | 1.7 | Proteobacteria; Alphaproteobacteria; Rhodobacterales; Rhodobacteraceae; Roseovarius |
| Otu7 | 0.0 | 0.2 | 13.7 | 3.4 | Bacteroidetes; Cytophagia; Cytophagales; Flammeovirgaceae; Fabibacter |
| Otu8 | 0.0 | 0.0 | 0.3 | 16.0 | Bacteroidetes; Cytophagia; Order_III; Unknown_Family; Fodinibius |
| Otu9 | 7.8 | 0.0 | 0.1 | 0.0 | Bacteroidetes; Flavobacteriia; Flavobacteriales; Flavobacteriaceae; Chryseobacterium |
| Otu10 | 0.0 | 0.0 | 0.4 | 14.7 | Bacteroidetes; Cytophagia; Order_III; Unknown_Family; unclassified |
| Otu11 | 0.0 | 13.4 | 0.2 | 0.0 | Proteobacteria; Gammaproteobacteria; Alteromonadales; Alteromonadaceae; Alteromonas |
| Otu12 | 7.4 | 0.0 | 0.0 | 0.0 | Proteobacteria; Alphaproteobacteria; Sphingomonadales; Sphingomonadaceae; Sphingomonas |
| Otu13 | 3.1 | 4.7 | 0.0 | 0.0 | Proteobacteria; Gammaproteobacteria; Alteromonadales; Alteromonadaceae; Marinobacter |
| Otu14 | 5.4 | 0.0 | 0.0 | 0.0 | Proteobacteria; Alphaproteobacteria; Sphingomonadales; Sphingomonadaceae; Sphingobium |
| Otu15 | 4.5 | 0.0 | 0.0 | 0.0 | Proteobacteria; Betaproteobacteria; Rhodocyclales; Rhodocyclaceae; Zoogloea |
| Otu16 | 0.0 | 7.6 | 0.0 | 0.0 | Proteobacteria; Gammaproteobacteria; Oceanospirillales; Oceanospirillaceae; Marinomonas |
| Otu18 | 0.1 | 0.5 | 0.8 | 3.0 | Bacteroidetes; Flavobacteriia; Flavobacteriales; Flavobacteriaceae; Salegentibacter |
| Otu1 | - | 33.9 | 22.4 | 18.6 | Euryarchaeota; Halobacteria; Halobacteriales; Halobacteriaceae; Halorubrum |
| Otu2 | - | 14.2 | 11.4 | 13.2 | Euryarchaeota; Halobacteria; Halobacteriales; Halobacteriaceae; Halorubrum |
| Otu3 | - | 0.0 | 9.5 | 8.0 | Euryarchaeota; Halobacteria; Halobacteriales; Halobacteriaceae; Halolamina |
| Otu4 | - | 12.1 | 7.0 | 6.9 | Euryarchaeota; Halobacteria; Halobacteriales; Halobacteriaceae; Halolamina |
| Otu5 | - | 0.0 | 6.8 | 9.8 | Euryarchaeota; Halobacteria; Halobacteriales; Halobacteriaceae; Halobacterium |
| Otu6 | - | 19.9 | 5.4 | 4.0 | Euryarchaeota; Halobacteria; Halobacteriales; Halobacteriaceae; Halohasta |
| Otu7 | - | 17.5 | 2.0 | 3.8 | Euryarchaeota; Halobacteria; Halobacteriales; Halobacteriaceae; Halonotius |
| Otu8 | - | 0.0 | 4.8 | 5.9 | Euryarchaeota; Halobacteria; Halobacteriales; Halobacteriaceae; Natronomonas |
