# Supplementary figures and images for: Changes in bacterial and archaeal communities during the concentration of brine at the graduation towers in Ciechocinek spa (Poland)
Source: Extremophiles. 2017 Dec 19;22(2):233–46. doi: 10.1007/s00792-017-0992-5 (PMC5847177; doi:10.1007/s00792-017-0992-5)

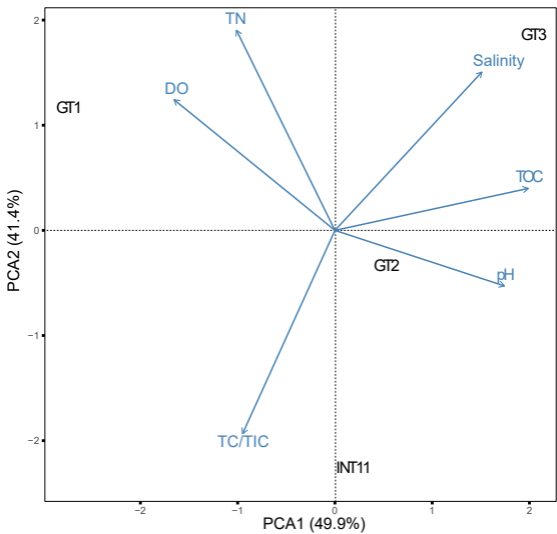

Supplement: Supplementary file 2 — Fig. S1 PCA of physical-chemical properties of brines from the system of graduation towers in Ciechocinek (PDF 27 kb) [file 792_2017_992_MOESM2_ESM.pdf]

A

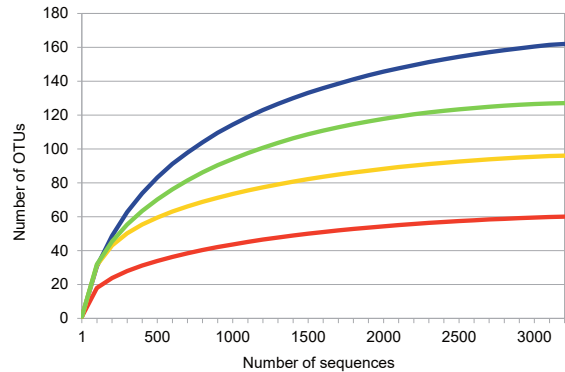

B

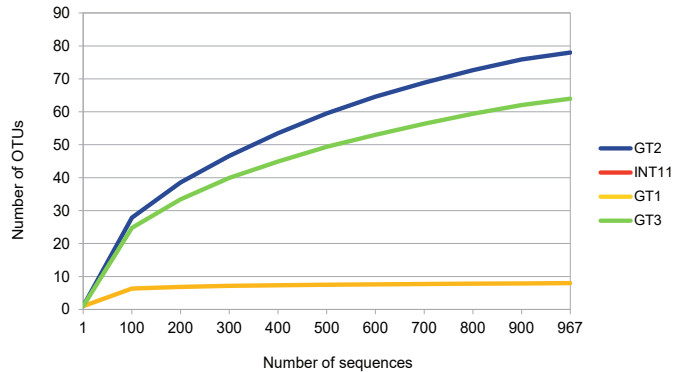

Supplement: Supplementary file 3 — Fig. S2 Rarefaction analysis of sequences from the brines from Ciechocinek at 0.03 dissimilarity level; a - bacterial b - archaeal INT11 – sample collected from the borehole, GT1-GT3 – bottom tank samples collected from the three graduation towers (PDF 51 kb) [file 792_2017_992_MOESM3_ESM.pdf]

A

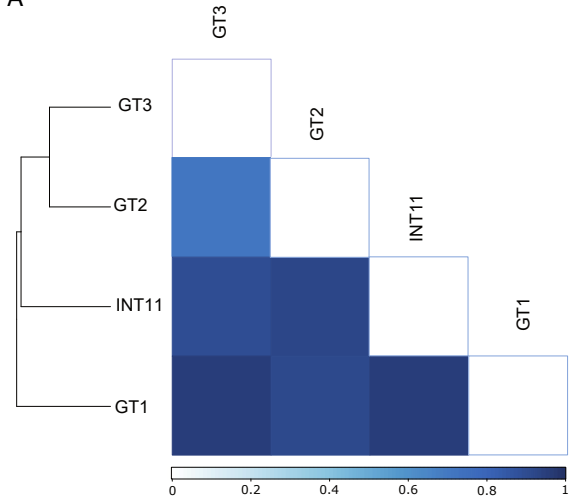

B

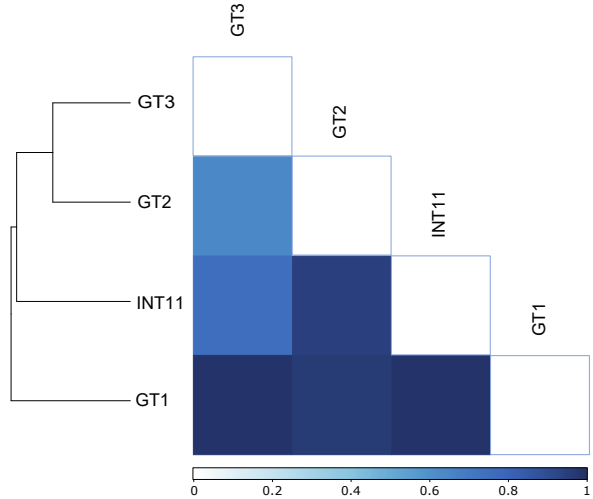

Supplement: Supplementary file 4 — Fig. S3 Bacterial community distance heatmaps. Heatmaps based on Bray-Curtis (A) and Morisita-Horn (B) at 0.03 dissimilarity level. Light blue - small distances (greater similarity), dark blue - greater distances (lower similarity) (PDF 21 kb) [file 792_2017_992_MOESM4_ESM.pdf]

A

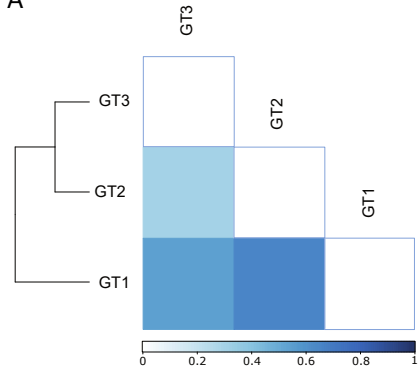

B

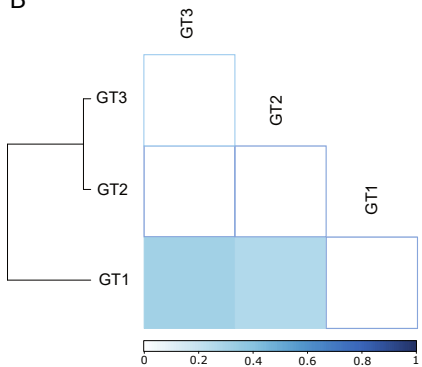

Supplement: Supplementary file 5 — Fig. S4 Archaeal community distance heatmaps. Heatmaps based on Bray-Curtis (A) and Morisita-Horn (B) at 0.03 dissimilarity level. Light blue - smaller distances (greater similarity), dark blue - greater distances (lower similarity) (PDF 20 kb) [file 792_2017_992_MOESM5_ESM.pdf]
